# Combination versus single-agent as palliative chemotherapy for gastric cancer

**DOI:** 10.1186/s12885-020-6666-1

**Published:** 2020-03-02

**Authors:** Jin-Hyuk Choi, Yong Won Choi, Seok Yun Kang, Geum Sook Jeong, Hyun Woo Lee, Seong Hyun Jeong, Joon Seong Park, Mi Sun Ahn, Seung Soo Sheen

**Affiliations:** 1 Department of Hematology-Oncology, Ajou University School of Medicine, 164 World Cup-ro, Suwon, Yeongtong-gu 16499 South Korea; 2 Department of Pulmonary and Critical Care Medicine, Ajou University School of Medicine, Suwon, South Korea

**Keywords:** Gastric cancer, Palliative chemotherapy, Single, Combination, Platelet-to-lymphocyte ratio, Age

## Abstract

**Background:**

Although combination chemotherapy (CC) is generally recommended in recurrent or primary metastatic gastric cancer (RPMGC), the results of randomized trials are conflicting.

**Methods:**

A retrospective review was conducted on 687 RPMGC patients who received palliative chemotherapy. We compared the overall survival (OS) between CC and single-agent chemotherapy (SC) among these patients, and we analyzed the clinicopathological characteristics affecting outcome including neutrophil-to-lymphocyte ratio (NLR) and platelet-to-lymphocyte ratio (PLR).

**Results:**

Although 521 patients (75.8%) underwent CC, SC was more frequently performed in elderly patients (57.6%) and ECOG performance status (PS) 2 or 3 (65.8%) patients (*p* < 0.0001, in each case). The median OS of patients who received CC was significantly longer than that of patients who received SC (11 vs. 8 months, *p* < 0.0001). No difference in OS between CC and SC was observed in elderly patients (*p* = 0.583), poor PS (*p* = 0.810), signet ring cell (*p* = 0.347), palliative surgical resection (*p* = 0.307), and high PLR (*p* = 0.120), with a significant interaction between age and type of regimen (*p* = 0.012). Moreover, there was no difference in OS between CC and SC after propensity score matching (*p* = 0.322). Multivariate analysis revealed that palliative resection and ≥ second-line chemotherapy were independently associated with favorable OS (*p* < 0.0001, in each case), whereas poor PS (*p* = 0.004), signet ring cell (*p* < 0.0001), peritoneal metastasis (*p* = 0.04), high NLR (*p* = 0.001), and high PLR (*p* = 0.033) were independent prognostic factors of poor OS.

**Conclusions:**

Although CC is the standard of care in RPMGC, SC can be considered a reasonable option in certain subgroups, such as elderly patients.

## Background

Gastric cancer (GC) is the most common malignancy in Korea and the second leading cause of cancer-related death worldwide [1, 2]. For patients with recurrent or primary metastatic GC (RPMGC), palliative chemotherapy is the standard of care. In terms of chemotherapy regimen, combination chemotherapy (CC) is generally recommended in clinical practice [3–7]. Two meta-analyses demonstrated a small but statistically significant survival benefit of CC compared to single-agent chemotherapy (SC) [6, 7]. However, individual randomized trials comparing CC and SC gave conflicting results [4, 6–10]. Moreover, most randomized trials excluded patients who were elderly or who had poor performance status (PS) [3–5, 8–11]. Therefore, it is an important clinical issue to determine clearly whether CC is more beneficial than SC and to identify patient subgroups who may benefit from SC rather than CC.

There is increasing evidence that inflammation plays a critical role in the development and progression of cancers [12–14]. Many inflammation-based prognostic markers have been suggested as potential prognostic factors in various types of cancers [12–14]. Recently, as convenient and cost-effective blood-derived markers, the neutrophil-to-lymphocyte ratio (NLR) and the platelet-to-lymphocyte ratio (PLR), which may reflect the inflammatory response, immune response, and coagulation status, have been widely investigated as useful prognostic factors in many solid tumors including GC [12–16].

In this study, we compared the overall survival (OS) between CC and SC in RPMGC patients, while analyzing the clinicopathological characteristics affecting outcome including NLR and PLR.

## Methods

### Study population

All histologically documented RPMGC patients who had started first-line palliative chemotherapy at Ajou University Hospital between January 2004 and December 2014 were retrospectively identified. Histologically documented case of RPMGC were eligible. In patients with primary metastatic disease, American Joint Committee on Cancer stage IV patients [17] with distant metastasis were included. Definition of primary metastatic disease in patients with surgical resection before chemotherapy was previously described [18]. Patients who had started first-line chemotherapy at other hospitals during this period and received further therapy at our institution were excluded.

All procedures performed in the study involving human participants were carried out in accordance with the ethical standards of the institutional and/or national research committee and with the 1964 Helsinki Declaration and its later amendments or comparable ethical standards. The protocol was reviewed and approved by the Institutional Review Board (IRB) of Ajou University Hospital (IRB approval no. AJIRB-MED-MDB-18-317). The IRB decided to waive the informed consent for this study because it was a retrospective study using anonymized data. Studies about third or further line of chemotherapy and palliative surgical resection, which included the majority of the patients of the present study cohort, were previously reported [18, 19]. However, the eligibility criteria of the current study were somewhat different from those of the previous ones with longer follow-up of patients [18, 19].

### Clinical review

We retrospectively reviewed the medical records of the eligible patients. Data collected on the RPMGC patients included various clinicopathological characteristics of patients and survival information. Pathologic information on the primary tumor of the stomach in both primary metastatic and recurrent disease was used for histologic subclassification, while histology was classified according to the pathology report on the recurrent stomach lesion, if available, in local recurrence cases.

Complete blood count (CBC) with differential count obtained just before first line chemotherapy was used to determine NLR and PLR. The NLR and PLR were calculated from the differential count by dividing the neutrophil or platelet count by the lymphocyte count. NLR or PLR greater than median values were defined a prior as high level.

### Statistical analysis

OS was calculated using the Kaplan–Meier method. OS was defined as the time from the start day of first-line chemotherapy to death. Data on the survivors were censored at the last follow-up. The log-rank test was used for analysis of the differences between the survival curves. The comparison of the categorical variables between groups was performed by Fisher’s exact test. The Cox proportional hazards regression model was applied to determine the joint effects of several variables on survival and to assess interactions between treatment and subgroups in subgroup analyses. In the Cox proportional hazards regression model, factors with *p* values < 0.1 in univariate analysis were included. All statistical analyses were performed two-sided using SPSS version 23.0 for Windows.

Propensity score matching (PSM) was applied to reduce selection bias by balancing covariates that may be associated with the outcome. In the current study, the 1:1 nearest neighbor matching was performed using SPSS version 23.0 for Windows.

## Results

### Patient characteristics

Of the 692 patients who started first-line palliative chemotherapy at our institution for RPMGC, five patients without CBC data before first-line chemotherapy were excluded, leaving 687 patients for analysis.

Table 1 summarizes the patients’ clinicopathological characteristics. Of the 687 patients, 478 (69.6%) were male, 125 (18.2%) were 70 years or older, with a median age of 57 (19–86), 611 (88.9%) were in ECOG PS 0 or 1, and 186 (27.1%) had poorly differentiated adenocarcinoma as the most prevalent histological type. A total of 314 (45.7%) and 152 (22.1%) patients had peritoneal and liver metastasis, respectively, and 32 (4.7%) patients had both liver and peritoneal metastases. Of the 304 patients with recurrent disease, 274 had received adjuvant chemotherapy. Palliative surgical resection (gastrectomy: 82; metastasectomy: 42; both: 14) before first-line therapy was performed in 138 patients (primary metastatic: 96, recurrent: 42). Among them, 60 patients underwent complete resection of tumor lesion(s) without gross residual disease. The reasons for performing palliative surgical resection before chemotherapy were previous described [19]. All the patients with primary metastatic disease were in AJCC stage IV, except for two stage III patients with gross residual disease after resection. Seven patients with primary metastatic disease who had undergone complete resection of the primary tumor with regional lymph node dissection had positive tumor cells in peritoneal cytology only.

**Table 1:** Patients characteristics at the initiation of first-line chemotherapy

Characteristics	Before propensity score matching			*P* value	After propensity score matching			*P* value
	Total *N* (%)	Chemotherapy			Total *N* (%)	Chemotherapy		
		Single-agent	Combination			Single-agent	Combination	
Gender				0.101				0.672
Male	478 (69.6)	107(64.5)	371 (71.2)		164 (69.5)	80(67.8)	84 (71.2)	
Female	209 (30.4)	59 (35.5)	150 (28.8)		72 (30.5)	38 (32.2)	34 (28.8)	
Age (years)				< 0.0001				0.88
< 70	562 (81.8)	94 (56.6)	468 (89.8)		160 (67.8)	79 (66.9)	81 (68.6)	9
≥ 70	125 (18.2)	72 (43.4)	53 (10.2)		76 (32.2)	39 (33.1)	37 (31.4)	
PS (ECOG)				< 0.0001				0.865
0, 1	611 (88.9)	116 (69.9)	495 (95.0)		194 (82.2)	98 (83.1)	96 (81.4)	
2, 3	76 (11.1)^a^	50 (30.1)	26 (5.0)		42 (17.8)^a^	20 (16.9)	22 (18.6)	
Disease status				0.325				0.696
Primary metastatic	383 (55.7)	87 (52.4)	296 (56.8)		116 (49.2)	56 (47.5)	60 (56.8)	
Recurrent	304 (44.3)	79 (47.6)	225 (43.2)		120 (50.8)	62 (52.5)	58 (49.2)	
Tumor differentiation				0.111				0.933
Well, moderate	167 (24.3)	50 (30.1)	117 (22.5)		67 (28.4)	34 (28.8)	33 (28.0)	
Poor	186 (27.1)	43 (25.9)	143 (27.4)		61 (25.8)	30 (25.4)	31 (26.3)	
Signet ring cell	171 (24.9)	32 (19.3)	139 (26.7)		59 (25.0)	28 (23.7)	31 (26.3)	
Combined, others	163 (23.7)	41 (24.7)	122 (23.4)		49 (20.8)	26 (22.0)	23 (19.5)	
Peritoneal metastasis				0.003				0.686
No	373 (54.3)	107 (64.5)	266 (51.1)		148 (62.7)	72 (61.0)	76 (64.4)	
Yes	314 (45.7)	59 (35.5)	255 (48.9)		88 (37.3)	46 (39.0)	42 (35.6)	
Liver metastasis				0.915				0.382
No	535 (77.9)	130 (78.3)	405 (77.7)		171 (72.5)	89 (75.4)	82 (69.5)	
Yes	152 (22.1)	36 (21.7)	116 (22.3)		65 (27.5)	29 (24.6)	36 (30.5)	
Palliative surgical resection				0.182				0.731
No	549 (79.9)	139 (83.7)	410 (78.7)		195 (82.6)	96 (81.4)	99 (83.9)	
Yes	138 (20.1)	27(16.3)	111 (21.3)		41 (17.4)	22 (18.6)	19 (16.1)	

^a^ PS 3: 2 patients

*N* number, *PS* performance status, *ECOG* Eastern Cooperative Oncology Group

First-line chemotherapy was combination therapy for 521 patients (75.8%) and single-agent therapy for 166 patients (24.2%). CC included: 5-FU/leucovorin/oxaliplatin (359 patients), S1/cisplatin (74), capecitabine/oxaliplatin (22), capecitabine or 5-FU/cisplatin/trastuzumab (9), and others (57). SC included S1 (146 patients) and others (20). SC was more frequently performed in older patients (≥70 years) (*p* < 0.0001) and in patients with poor PS (*p* < 0.0001), while a higher proportion of patients with peritoneal metastasis received CC (*p* = 0.003) (Table 1). For PSM, clinicopathological characteristics at the start of first-line chemotherapy were used as covariates, which were well balanced after 1:1 PSM (Table 1).

### Clinicopathological characteristics according to NLR and PLR

Table 2 summarizes the patients’ clinicopathological characteristics according to NLR and PLR. The median NLR and PLR were 2.67 (0.54–24.5) and 167.21 (10.53–851.44), respectively. High NLR and PLR were both associated with a high proportion of primary metastatic disease (*p* < 0.0001, in each case) and peritoneal metastasis (*p* = 0.039 and *p* < 0.0001, respectively). While high NLR was associated with a high proportion of poor PS (*p* = 0.02) and no palliative resection (*p* < 0.0001), high PLR was associated with a high proportion of absence of liver metastasis (*p* = 0.004). NLR and PLR correlated with each other significantly (*p* < 0.0001).

**Table 2:** Patients characteristics according to neutrophil-to-lymphocyte ratio /platelet-to-lymphocyte ratio

Characteristics	Total *N* (%)	NLR		*P* value	PLR		*P* value
		Low	High^a^		Low	High^b^	
Gender				0.116			0.562
Male	478 (69.6)	229 (66.8)	249 (72.4)		243 (70.6)	235 (68.5)	
Female	209 (30.4)	114 (33.2)	95 (27.6)		101 (29.4)	108 (31.5)	
Age (years)				0.375			0.235
< 70	562 (81.8)	276 (80.5)	286 (83.1)		275 (79.9)	287 (83.7)	
≥ 70	125 (18.2)	67 (19.5)	58 (16.9)		69 (20.1)	56 (16.3)	
PS (ECOG)				0.020			0.333
0, 1	611 (88.9)	315 (91.8)	296 (86.0)		310 (90.1)	301 (87.8)	
2, 3	76 (11.1) ^c^	28 (8.2)	48 (14.0)		34 (9.9)	42 (12.2)	
Disease status				< 0.0001			< 0.0001
Primary metastatic	383 (55.7)	155 (45.2)	228 (66.3)		166 (48.3)	217 (63.3)	
Recurrent	304 (44.3)	188 (54.8)	116 (33.7)		178 (51.7)	126 (36.7)	
Tumor differentiation				0.166			0.777
Well, moderate	167 (24.3)	92 (26.8)	75 (21.8)		88 (25.6)	79 (23.0)	
Poor	186 (27.1)	83 (24.2)	103 (29.9)		95 (27.6)	91 (26.5)	
Signet ring cell	171 (24.9)	81 (23.6)	90 (26.2)		81 (23.5)	90 (26.2)	
Combined, others	163 (23.7)	87 (25.4)	76 (22.1)		80 (23.3)	83 (24.2)	
Peritoneal metastasis				0.039			< 0.0001
No	373 (54.3)	200 (58.3)	173 (50.3)		220 (64.0)	153 (44.6)	
Yes	314 (45.7)	143 (41.7)	171(49.7)		124 (36.0)	190 (55.4)	
Liver metastasis				1.000			0.004
No	535 (77.9)	267 (77.8)	268 (77.9)		252 (73.3)	283 (82.5)	
Yes	152 (22.1)	76 (22.2)	76 (22.1)		92 (26.7)	60 (17.5)	
1st line CTx				0.286			0.929
Single-agent	166 (24.2)	89 (25.9)	77 (22.4)		84 (24.4)	82 (23.9)	
Combination	521 (75.8)	254 (74.1)	267 (77.6)		260 (75.6)	261 (76.1)	
Palliative surgical resection				< 0.0001			0.775
No	549 (79.9)	250 (72.9)	299 (86.9)		273 (79.4)	276 (80.5)	
Yes	138 (20.1)	93 (27.1)	45 (13.1)		71 (20.6)	67 (19.5)	
PLR				< 0.0001			–
Low	344 (50.1)	248 (72.3)	96 (27.9)		–	–	
High	343 (49.9)	95 (27.7)	248 (72.1)		–	–	

^a^ > median (2.67), ^b^ > median (167.21). ^c^ PS 3: 2 patients

*NLR* neutrophil-to-lymphocyte ratio, *PLR* platelet-to-lymphocyte ratio, ***N*** number, *PS* performance status, *ECOG* Eastern Cooperative Oncology Group, *CTx* Chemotherapy

### Overall survival

**Fig. 1:**
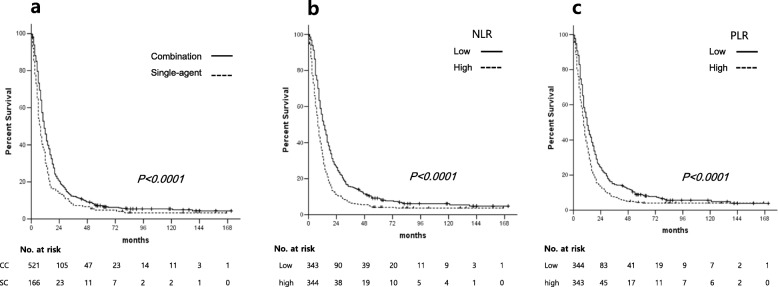
Overall survival according to **a** the type of regimen (combination vs. single), **b** NLR, and **c** PLR

The median follow-up duration for the survivors was 85 months (43–171 months). Only one patient was lost to follow-up at 1 month after the initiation of first-line chemotherapy, while included in survival analysis as censored data. At the time of the last follow-up, 35 patients (5.1%) were still alive. The median OS of all patients after initiation of first-line therapy was 10 months. The median OS of patients who received first-line CC was significantly longer than that of the patients who received SC (11 vs. 8 months, *p* < 0.0001) (Fig. 1a).

**Fig. 2:**
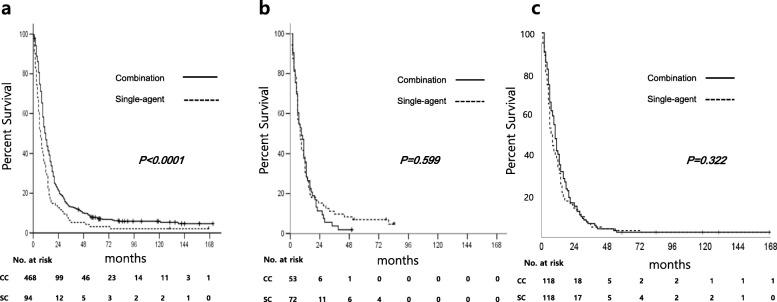
Overall survival according to the type of regimen (single vs. combination) in patients **a** < 70 years, **b** ≥ 70 years, and **c** after propensity score matching

**Fig. 3:**
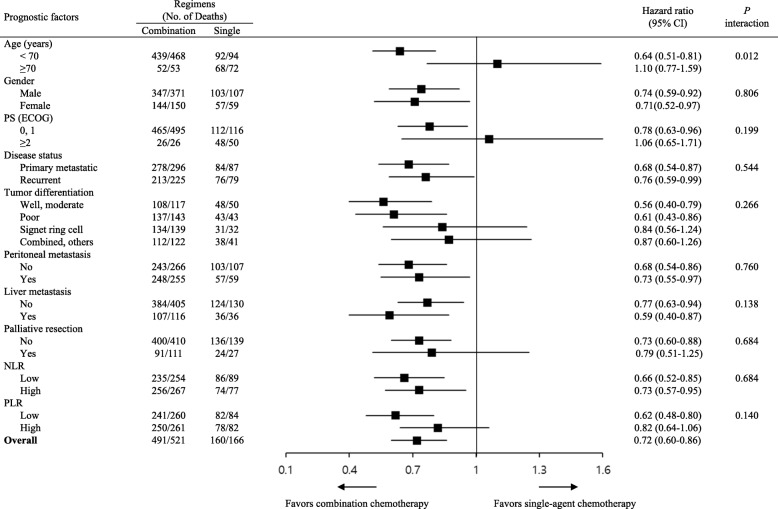
Forest plot for subgroup analyses of overall survival: the effect of regimens according to baseline characteristics. *CI*: confidence interval, *PS*: performance status; *ECOG*: Eastern Cooperative Oncology Group, *NLR*: neutrophil-to-lymphocyte ratio, *PLR*: platelet-to-lymphocyte ratio

In univariate analysis, patients who underwent surgical resection before first-line chemotherapy (*p* < 0.0001) and second- or further-line therapy (*p* < 0.0001) demonstrated longer median OS, as in first-line CC. Old age (70 years or older) (*p* = 0.012), poor PS (*p* < 0.0001), signet ring cell histology (*p* = 0.022), presence of peritoneal metastasis (*p* = 0.001), high NLR (*p* < 0.0001), and high PLR (*p* < 0.0001) were associated with poor OS (Table 3, Figs. 1b, c). Although patients with CC showed better OS in the majority of subgroups, no difference in OS between CC and SC were observed in patients with old age (*p* = 0.583, Fig. 2b), ECOG PS 2 or 3 (*p* = 0.810), signet ring cell and combined/other histology (*p* = 0.347 and *p* = 0.451, respectively), palliative surgical resection (*p* = 0.307), and high PLR (*p* = 0.120) (Fig. 3). There was a significant interaction between age and type of regimen (CC vs. SC) (*p* = 0.012) (Fig. 3).

**Table 3:** Univariate and multivariate analysis of overall survival from the start of first-line chemotherapy

Prognostic Factors	Before propensity score matching					After propensity score matching				
	Univariate		Multivariate			Univariate		Multivariate		
	MS (months)	*P* value^a^	HR	95% CI	*P* value^b^	MS (months)	*P* value^a^	HR	95% CI	*P* value^b^
Gender		0.728					0.688			
Male	10					9				
Female	10					8				
Age (years)		0.012					0.565			
< 70	11		1			9				
≥ 70	8		1.01	0.80–1.27	0.941	9				
PS (ECOG)		< 0.0001					< 0.0001			
0, 1	11		1	1		10		1	1	
≥ 2	5		1.47	1.13–1.92	0.004	5		1.59	1.10–2.28	0.013
Disease status		0.315					0.336			
Primary metastatic	10					8				
Recurrent	10					10				
Tumor differentiation		0.022					0.215			
Well, moderate	12		1			11				
Poor	10		1.32	1.06–1.65	0.015	9				
Signet ring cell	8		1.64	1.30–2.07	< 0.0001	7				
Combined, others	11		1.21	0.96–1.53	0.102	12				
Peritoneal metastasis		0.001					0.037			
No	11		1			10		1		
Yes	9		1.19	1.01–1.40	0.040	7		1.25	0.96–1.64	0.099
Liver metastasis		0.595					0.613			
No	11					9				
Yes	9					9				
Palliative resection		< 0.0001					< 0.0001			
No	9		1			8		1		
Yes	19		0.42	0.34–0.52	< 0.0001	15		0.59	0.41–0.85	0.005
1st line CTx		< 0.0001					0.322			
Single-agent	8		1			8				
Combination	11		0.82	0.66–1.02	0.069	10				
CTx lines		< 0.0001					< 0.0001			
1st line CTx	6		1			6		1		
≥ 2nd line CTx	14		0.69	0.59–0.82	< 0.0001	13		0.59	0.45–0.77	< 0.0001
NLR		< 0.0001					0.013			
Low	13		1			10		1		
High	8		1.35	1.14–1.61	0.001	7		1.25	0.95–1.65	0.111
PLR		< 0.0001					0.539			
Low	12		1			9				
High	9		1.21	1.02–1.44	0.033	8				

^a^ Log-rank test, ^b^Cox proportional-hazards regression model

*MS* median survival, *HR* hazard ratio, *PS* performance status, *ECOG* Eastern Cooperative Oncology Group, *CTx* chemotherapy, NLR neutrophil-to-lymphocyte ratio, *PLR* platelet-to-lymphocyte ratio

Multivariate analysis revealed that palliative resection and ≥ second-line chemotherapy were independently associated with favorable OS (*p* < 0.0001, in each case), whereas ECOG PS 2 or 3 (*p* = 0.004), poorly differentiated and signet ring cell histology (*p* = 0.015 and *p* < 0.0001, respectively), peritoneal metastasis (*p* = 0.04), high NLR (*p* = 0.001), and high PLR (*p* = 0.033) were independent prognostic factors of poor OS (Table 3). CC was not independently associated with favorable OS (Table 3). Given the strong correlation between NLR and PLR, we performed multivariate analysis that included only one of the two on each occasion. Both high NLR and high PLR were independent prognostic factor of poor OS in these analyses (*p* < 0.0001, in each case, detailed data not shown).

After PSM, there was no significant difference in OS between CC and SC (*p* = 0.322), while poor PS, palliative resection, and ≥ second-line chemotherapy still demonstrated independent prognostic significance (Fig. 2c and Table 3). In addition, no significant difference in OS between CC and SC was also observed in old age (*p* = 0.348) and ECOG 2 or 3 (*p* = 0.461) patients in the PSM cohort.

## Discussion

In the present study, patients with old age or poor PS patients underwent SC more frequently despite the significantly higher proportion of RPMGC patients with CC. On the other hand, CC was more commonly used in patients with peritoneal metastases. The most likely explanation for these findings is that oncologists judged that CC was too toxic for elderly patients and those with poor PS, whereas patients with peritoneal metastases required aggressive treatment, given its association with poor outcome. Although CC demonstrated an OS benefit in univariate analysis, it was not associated with favorable outcome in several subgroups such as elderly patients, and had no prognostic significance in multivariate analysis.

Large phase III trials to compare CC and SC incorporating third generation agents, such as S-1, docetaxel and irinotecan, have been conducted in Japan and have shown conflicting results [4, 8–10]. In two trials, adding cisplatin or docetaxel to S-1 showed an OS benefit over S-1 alone [9, 10]. However, no significant difference in OS was observed between CC and S-1 monotherapy in two other trials [4, 8]. A recent meta-analysis of chemotherapy in advanced gastric cancer indicated that survival was significantly but slightly improved (about 1 month) with CC compared to SC [7]. Only a few retrospective studies have compared the outcomes between CC and SC. Two large retrospective studies demonstrated that CC was superior to SC in terms of OS [20, 21]. However, unlike the present study, neither retrospective study investigated the relationship between chemotherapy regimen and important clinical characteristics such as age, PS, and palliative resection [20, 21]. Moreover, because most randomized trials have included relatively young patients and patients with good PS, it is difficult to define the best chemotherapy strategy for elderly patients or those with poor PS [3–5, 8–11].

In the present study, no difference in OS between CC and SC was observed in patients with old age, ECOG PS 2 or 3, signet ring cell and combined/other histology, palliative surgical resection, and high PLR. Despite a lack of significant interaction between ECOG PS and type of regimen probably due to the small number of patients with PS 2 or 3, SC showed similar OS compared to CC. Given the relatively high risk of chemotherapy-related toxicities in CC, SC could be recommended in clinical practice for patients with poor PS. In patients with palliative surgical resection before first-line chemotherapy, there was no significant difference in OS between the CC and SC groups. Low tumor burden after palliative resection may be related to this result. However, routine use of SC in patients with palliative resection before chemotherapy cannot be recommended on the basis of these findings, given the retrospective nature of the present study, without significant interaction between surgical resection and chemotherapy regimen.

Limited data are available regarding chemotherapy regimens for elderly patients with RPMGC, because such patients are underrepresented in clinical trials [3–5, 8–11]. Two small retrospective studies comparing S-1 with S-1 and cisplatin (SP) for RPMGC patients older than 70 gave conflicting results, with both studies showing more severe toxicities in the SP group [11, 22]. Two larger retrospective studies from Korea and Japan for elderly patients demonstrated no difference in OS between CC and SC [3, 5]. In one of these studies, there was no significant difference in OS between the S-1 and SP groups, even after propensity score matching [3]. Furthermore, in a small phase III trial from Korea, comparing capecitabine with capecitabine and oxaliplatin in 50 elderly (≥70 years) RPMGC patients, there was no significant difference in OS, with higher incidence of some toxicities in CC arm [23]. In the current study cohort of elderly patients (≥ 70 years), only 3.8% of the CC group were in PS 2, while 25% of the SC group were in PS 2 (*p* = 0.001). Despite a significantly higher proportion of patients with good PS in the CC group, there was no difference in OS between the two groups in elderly patients. This finding and the significant interaction between regimen (CC vs. SC) and age suggest that CC may not be beneficial compared to SC in elderly patients with RPMGC, especially those aged 70 years or more. Given the results of the present study and previous investigations, SC can be recommended as a reasonable option for elderly patients, especially those with poor PS or comorbidity, although randomized trials are essential to define the standard chemotherapy regimen.

Considering imbalance in age and PS, well-established prognostic factors, between CC and SC groups, in the present cohort, PSM analysis was performed by adjusting patient characteristics before the initiation of chemotherapy as covariates. After PSM, there was no difference in OS between CC and SC groups. Increased proportion of patients with old age and ECOG PS 2 or 3 (10.2 to 31.4% and 5.0 to 18.6%, respectively) in CC group after PSM may be attributable to this result. In addition, no difference in OS between CC and SC was observed in old age and poor PS patients after PSM. These findings also suggest that SC could be useful option with less toxicity in elderly and poor PS patients.

Inflammation plays a critical role in the development and progression of various cancers [12–14]. Of the various inflammatory markers, NLR and PLR have been suggested as potential prognostic markers in various cancers [12–16]. A high NLR reflects a decrease in the number of lymphocytes and/or an elevated number of neutrophils. Neutrophils may play an important role in the development and progression of cancer by offering a suitable microenvironment for their growth [14, 24, 25]. Circulating neutrophils may secrete vascular endothelial growth factor (VEGF), interleukin-18, and matrix metalloproteinase, which are closely associated with tumorigenesis, progression and metastasis [14, 15, 24, 25]. Furthermore, the antitumor immune responses of activated T cells and natural killer cells may be inhibited by an elevated number of neutrophils surrounding tumor tissues [14, 24, 25]. Therefore, an elevated neutrophil count may have a negative effect on cancer patients, leading to poor outcome. In addition, because lymphocyte plays a crucial role in cellular adaptive immunity against cancer by attacking tumor cells at the outset of tumorigenesis, lymphopenia may reflect suppressed cell-mediated immunity against cancer [13–16].

An elevated level of PLR also represents an increased number of platelets and/or a decreased number of lymphocytes. Elevated platelet counts may promote the metastatic potential of tumor cells in various biological pathways [13, 16, 24]. Platelets may secrete cellular growth factors such as platelet-derived growth factor, VEGF, transforming growth factor beta, and platelet factor 4, thereby stimulating tumor angiogenesis and growth [13, 16, 24]. In addition, platelets can activate the invasiveness of tumor cells by enhancing the formation of tumor stroma and supporting the adhesion of tumor cells to the endothelium [13, 16]. Furthermore, in the bloodstream, interactions between tumor cells and platelets could facilitate tumor cell metastasis by impeding the clearance of tumor cells by immune cells [13, 16].

In RPMGC, several studies have reported a significant association between high NLR or PLR and poor OS in patients treated with palliative chemotherapy [12, 14, 15, 24–26]. In the present study, both NLR and PLR were independently associated with poor OS in RPMGC patients who received palliative chemotherapy. NLR correlated significantly with PS and palliative resection, well-established prognostic factors in RPMGC, but there was no significant association between PLR and the same factors.

One interesting finding in the current study is that there was no significant difference in OS between CC and SC in the high PLR group, despite the lack of interaction between PLR and regimen. A possible explanation for similar OS between CC and SC in the high PLR group is that high platelet counts reflect aggressive behavior of tumors that are refractory even to CC. Alternatively, CC may decrease lymphocyte count more than SC, leading to greatly suppressed cell-mediated immunity against cancer cells. Chemotherapy-induced lymphopenia is commonly observed event, especially in dose-dense regimens [27, 28]. Moreover, a few studies showed significant association between lymphopenia after chemotherapy with or without radiotherapy and poor outcome in several solid tumors [27, 29].

Having analyzed a relatively large number of patients, the present study reported comparable OS between CC and SC in certain subgroups of RPMGC patients, which might provide useful information for clinical decision-making. However, the current study has several limitations. First, it is a retrospective analysis from a single institution. Second, a variety of chemotherapy regimens were used in several therapy lines. Third, because the data were not prospectively collected, we did not analyze chemotherapy-related toxicities. Fourth, a very small number of patients were treated with first-line trastuzumab containing regimen, owing to the approval time of trastuzumab in Korea. Finally, because the optimal cut-off values of NLR and PLR have not yet been determined, application of the data from the present study to clinical practice requires further validation [14, 16]. Nonetheless, because the present study analyzed all patients who underwent palliative chemotherapy during the defined period with mature follow-up (minimum follow-up duration of survivors: 43 months), the results may reflect treatment outcomes in real-world clinical practice.

## Conclusion

Although CC is the standard of care in RPMGC, SC can be considered a reasonable option in certain subgroups, such as elderly patients.

## Data Availability

It is regrettable that we will not be able to provide the raw data of the present study. Under the current South Korean law (Bioethics and Safety Act), data of human subjects including personal information, even in de-identified form, can be provided to a third party only after obtaining a written consent from the subject. Because the present study is retrospective one about palliative chemotherapy for advanced cancer, it is practically impossible to get written consents from the patients at this point.

## Abbreviations

CBC: Complete blood count
CC: Combination chemotherapy
ECOG: Eastern Cooperative Oncology Group
GC: Gastric cancer
IRB: Institutional Review Board
NLR: Neutrophil-to-lymphocyte ratio
OS: Overall survival
PLR: Platelet-to-lymphocyte ratio
PS: Performance status
PSM: Propensity score matching
RPMGC: Recurrent or primary metastatic gastric cancer
SC: Single-agent chemotherapy
SP: S-1 and cisplatin
VEGF: Vascular endothelial growth factor
